# Detection of *Babesia annae* DNA in lung exudate samples from Red foxes (*Vulpes vulpes*) in Great Britain

**DOI:** 10.1186/s13071-016-1364-1

**Published:** 2016-02-12

**Authors:** Paul M. Bartley, Clare Hamilton, Cari Wilson, Elisabeth A. Innes, Frank Katzer

**Affiliations:** Moredun Research Institute, Pentlands Science Park, Bush Loan, Midlothian, EH26 0PZ Scotland UK

**Keywords:** *Babesia annae*, DNA, Red foxes, *Vulpes vulpes*, Babesiosis, Great Britain, Phylogeny

## Abstract

**Background:**

This study aimed to determine the prevalence of *Babesia* species DNA in lung exudate samples collected from red foxes (*Vulpes vulpes*) from across Great Britain. *Babesia* are small piroplasmid parasites which are mainly transmitted through the bite of infected ticks of the family Ixodidae. *Babesia* can cause potentially fatal disease in a wide-range of mammalian species including humans, dogs and cattle, making them of significant economic importance to both the medical and veterinary fields.

**Methods:**

DNA was extracted from lung exudate samples of 316 foxes. A semi-nested PCR was used to initially screen samples, using universal *Babesia*-*Theileria* primers which target the 18S rRNA gene. A selection of positive PCR amplicons were purified and sequenced. Subsequently specific primers were designed to detect *Babesia annae* and used to screen all 316 DNA samples. Randomly selected positive samples were purified and sequenced (GenBank accession KT580786). Clones spanning a 1717 bp region of the 18S rRNA gene were generated from 2 positive samples, the resultant consensus sequence was submitted to GenBank (KT580785). Sequence KT580785 was used in the phylogenetic analysis

**Results:**

*Babesia annae* DNA was detected in the fox samples, in total 46/316 (14.6 %) of samples tested positive for the presence of *Babesia annae* DNA. The central region of England had the highest prevalence at 36.7 %, while no positive samples were found from Wales, though only 12 samples were tested from this region. Male foxes were found to have a higher prevalence of *Babesia annae* DNA than females in all regions of Britain. Phylogenetic and sequence analysis of the GenBank submissions (Accession numbers KT580785 and KT580786) showed 100 % identity to *Babesia* sp.-‘Spanish Dog’ (AY534602, EU583387 and AF188001).

**Conclusions:**

This is the first time that *Babesia annae* DNA has been reported in red foxes in Great Britain with positive samples being found across England and Scotland indicating that this parasite is well established within the red fox population of Britain. Phylogenetic analysis demonstrated that though *B. annae* is closely related to *B. microti* it is a distinct species.

## Background

*Babesia* are small piroplasmid parasites which are widely distributed throughout the world and are transmitted to hosts through the bite of infected ticks, transplacentally [1] and mechanically through the exchange of blood i.e., in dog fights [2] as well as transovarially and transstadially in ticks . More than 100 species of *Babesia* have been documented [3], *Babesia* parasites are capable of infecting a wide range of wild and domestic host species, including humans, cattle and dogs. This ability to infect and cause disease in many mammalian species make *Babesia* of great economic importance in both the medical and veterinary fields. Bovine babesiosis (Red water fever) for example is considered the most important arthropod transmitted disease in cattle [4]. In Europe the main cause of human babesiosis is considered to be *B. divergens* [4], though over recent years other *Babesia* species including *B. microti* [5] have also been found to be responsible for human babesiosis. *Babesia annae* is among these economically important species having been shown to cause severe (even fatal) disease in dogs [1, 2].

Little is known about the prevalence of piroplasm infections in dogs in Great Britain. A recent study by Crawford and colleagues [6] examining 262 canine blood donors found none were positive for Babesia DNA, while a study by Smith and colleagues [7] found only 2.4 % 16/742 ticks collected from dogs from across Great Britain tested PCR positive for *Babesia* DNA, 11 of which showed 97–100 % identity to *B. gibsoni*. There are no records of *Babesia annae* having been previously detected in Britain.

*Babesia annae* was identified previously under numerous synonyms including *Theileria annae*, *Babesia* sp. ‘Spanish dog’ and *Babesia-microti*-like [8]. This species has been found in red foxes (*Vulpes vulpes*), and dogs (*Canis familiaris*) in North America and Canada [9, 10] and throughout Europe [1, 11–15] and is considered by some authors to be “hyperendemic” in northwest Spain [16].

Red foxes are common throughout Great Britain, being highly adaptive and opportunistic predators and scavengers [17]. The most recent surveys suggested that there are between 240,000 and 258,000 red foxes in Britain, of which approximately 87 % (225,000) live in rural areas, compared to 13 % (33,000) of urban foxes (Game & Wildlife Conservation Trust (2013) and DEFRA FOI release (2013). The foxes are most likely to encounter questing adult ticks, nymphs and larvae whilst hunting and scavenging. Anthropogenic changes, such as wildlife and forest management strategies and changes in land use have also created more suitable habitats for foxes, their prey and the Ixodid ticks that feed on both. These changes have lead not only to increases in tick numbers, but have also enabled ticks to increase their distribution across Europe, with *Ixodes* ticks now being found in areas previously considered tick free i.e., northern Sweden [18].

The main tick species found in Britain are *Ixodes ricinus* (sheep tick, which is also known in some countries as the deer tick) and *Ixodes hexagonus* (hedgehog tick). Both tick species can be found on a wide range of domestic and wild animal species and have been shown in previous studies in Spain and Germany to be positive for *B. annae* (synonym *T. annae*) DNA [15, 19]. This is however not conclusive proof that these species are vectors for the transmission of *B. annae*.

This study aimed at determining the prevalence of piroplasm infection in red foxes and the species of piroplasm circulating in the red fox population in Great Britain, through the analysis of lung exudate samples.

## Methods

### Sample collection and preparation

The three hundred and sixteen foxes were originally collected by the University of Edinburgh as part of a study looking at the prevalence of *Echinococcus multilocularis, Neospora caninum and Toxoplasma gondii* [20, 21]. Lung samples were collected at post mortem examination and frozen at −20 °C prior to processing. Lung fluids were prepared as previously described [20]. Briefly, lungs were defrosted and bloody exudate was collected into a 1.5 ml microfuge tube, where no exudate was visible, lung samples were placed in a stomacher bag with approximately 5 ml of phosphate buffer saline (PBS) and processed in a stomacher for 15 s. All exudates / PBS samples were stored at −20 °C prior to DNA extraction.

The foxes sampled were collected over a wide range of locations in each of the study regions. A majority of the foxes were shot by game keepers and land owners as part of routine pest control procedures, so the location data from where the foxes were originally collected and the gender of the animals were often available. However some of the foxes were obtained from direct culls, for these animals the location and gender of the animal was not always available [21].

### DNA extraction

Three hundred and sixteen lung exudate samples were defrosted and mixed by vortexing, 400 μl of each sample was added to 900 μl Nuclei Lysis Solution (Promega, Madison WI, USA) and incubated at 55 °C overnight. The samples were then processed to DNA using the Wizard® genomic DNA (Promega, Madison WI, USA) purification protocol, which was adapted by Bartley and colleagues [22] to be scaled up to allow the use of 400 μl of starting material. The DNA was resuspended in 300 μl of DNase and RNase free water and stored at +4 °C for immediate use or at −20 °C for longer term storage. Extraction controls (water) were also prepared with each batch of exudate samples, these were used as indicators of contamination and acted as additional negative controls.

### PCR for detection of *Babesia* DNA in blood samples

A semi-nested PCR was used to screen for the presence of *Babesia* DNA in 120 lung exudate samples using universal *Babesia*-*Theileria* primers BT1-F and BTH-1R for the primary amplification and BT1-F and BT1-R for the second round amplification (previously described [23]). Following this initial screening the *B. annae* specific primers BTFox1F and BTFox1R were designed (Primer3web v4.0.0) for use in the second round amplifications (Table 1). All 316 lung exudate samples were tested using the *B. annae* specific primers BTFox1F and BTFox1R.

**Table 1:** Primer names, specificity and sequences used for the detection of *Babesia* DNA in fox lung exudate samples

Primer Name	Specificity^a^	Sequence (5’ – 3’)	Reference
BT1-F	Universal *Babesia* – *Theileria*	GGTTGATCCTGCCAGTAGT	[23]
BTH-1R	Universal *Babesia* – *Theileria*	TTGCGACCATACTCCCCCCA	
BTH-1 F	Universal *Babesia* – *Theileria*	CCTGMGARACGGCTACCACATCT	
BT1-R	Universal *Babesia* – *Theileria*	GCCTGCTGCCTTCCTTA	
BTFox1F	*Babesia annae*	AGTTATAAGCTTTTATACAGC	Developed in the study
BTFox1R	*Babesia annae*	CACTCTAGTTTTCTCAAAGTAAAATA	
BT-Outer-R	*Babesia annae*	GGAAACCTTGTTACGACTTCTC	
BT-Inner-R	*Babesia annae*	TTCTCCTTCCTTTAAGTGATAAG	
600-F	*Babesia annae*	AGTTAAGAAGCTCGTAGTTG	
1200-F	*Babesia annae*	AGGATTGACAGATTGATAGC	

^a^All primers were designed against the 18S rRNA gene

The reaction mixture was adapted from that previously described by Burrells and colleagues [24] and amplification conditions were as follows; each reaction (20 μl) consisted of custom PCR master mix (containing final concentrations of 45 mM Tris–HCl, 11 mM (NH_4_)_2_SO_4_, 4.5 mM MgCl_2_, 0.113 mg/ml BSA, 4.4 μM EDTA and 1.0 mM each of dATP, dCTP, dGTP and dTTP) (ABgene, Surrey, UK), 0.25pM of each forward and reverse primer (Eurofins MWG Operon), 0.75U *Taq* polymerase (Bioline Ltd. London, UK) and 2 μl sample template DNA, to increase sensitivity each sample was analysed in duplicate. The basic reaction conditions for all the PCR amplifications were as follows; 94 °C for 5 min followed by 35 cycles at 94 °C for 1 min, annealing (see Table 2) for 1 min and 72 °C for 1 min with a final extension period at 72 °C for 5 min.

**Table 2:** Annealing temperatures and amplicon sizes of nested PCR reactions used for the detection of *Babesia annae* DNA in fox lung exudate samples

Primers	Amplicon size (bp)	Annealing Temp (°C)
BT1-F and BTH-1R	1073	55
BT1-F and BT1-R	408	60
BTFox1F and BTFox1R	655	52
BT1-F and BT-Outer-R	1737	55
BT1-F and BT-Inner-R	1717	49

A selection of positive samples that gave positive results for both duplicates using the *B. annae* specific BTFox1F and BTFox1R primers were tested in a semi-nested PCR using the primers BT1-F and BT-Outer-R (Table 1) in the primary reaction and BT1-F and BT-Inner-R (Table 1) in the second round reaction. These primers were designed to produce a longer 18S rRNA gene fragment (approx 1.7Kb). Following the second round amplification, 10 μl of each PCR product was analysed by agarose gel electrophoresis (2 % agarose in 1x TAE buffer), stained with gel red (1:10,000) (Biotonium, Hayward, CA, USA) and visualised using UV light.

### PCR clean up and DNA sequencing

PCR products from samples that gave positive results for both duplicates using the BT1-F and BT-Outer-R (Table 1) in the primary reaction and BT1-F and BT-Inner-R primers in the second round reaction were selected for sequencing. These amplicons were cleaned using the commercially available Wizard® SV Gel and PCR Clean-up System (Promega, Madison WI, USA), as per manufacturers’ instructions. The PCR product was eluted in 30 μl of DNase / RNase free water and the nucleic acid concentration was determined by spectralphotometry (Nanodrop ND1000), 100 ng of each sample was sent for sequencing with each primer (MWG Operon) BT1-F, BTH-1 F, BTFox1F and BTFox1R (Table 1) to create a forward and reverse consensus sequence.

### Cloning of the 18 s rRNA gene of *Babesia annae*

Seven samples which gave positive results for both duplicates from the semi nested PCR (*B. annae* specific BT1-F and BT-Outer-R followed by BT1-F-BT-Inner-R), were selected for cloning and PCR products were purified as above, 7 μl of purified product was ligated into the pGEM®-T Easy Vector (Promega, Madison WI, USA) using 1 μl of T4 DNA ligase (3 Weiss units/μl), 1 μl of 10X Rapid Ligation Buffer and 1 μl of pGEM®-T Easy vector (50 ng/μl) according to the manufacturers’ instructions. Following ligation, 2 μl of ligated vector/insert was used to transform 50 μl of high-efficiency competent JM109 cells (≥1 × 10^8^ cfu/μg DNA) (Promega, Madison WI, USA) using manufacturers’ instructions, with the following exception that LB broth was used instead of SOC medium to culture the cells. Successful transformation was confirmed using LB agar plates containing 100 μg/ml ampicillin and spread with 100 μl of IPTG (100 mM) and 20 μl of X-Gal (50 mg/ml). White colonies were screened by PCR using the BTFox1F and BTFox1R primers (Table 1) to determine the presence of the *B. annae* 18S rRNA gene insert. Positive colonies were cultured overnight (approx 18 h) in 10 ml LB broth containing 100 μg/ml ampicillin. Following this incubation plasmid DNA was then purified from 5 ml of each culture using the QIAprep® Miniprep kit (Qiagen), according to the manufacturers’ instructions. Purified plasmid DNA (100 ng per primer) was sent to be sequenced (MWG Operon) using T7 and SP6 primers along with BTH-1 F, 600-F, 1200-F, BT1-R, and BTH-1R (Table 1), this created an overlapping forward and reverse consensus sequence for each clone.

### Sequence / phylogenetic analysis

Following sequencing, results were compared using NCBI Basic Local Alignment Search Tool (BLAST) to determine percentage identity of the generated sequences against published sequences. Multiple sequence alignments were created to compare these sequences to previously published data (EMBL-EBI Multiple Sequence Comparison by Log- Expectation (MUSCLE)), while phylogenetic analysis was performed on the long (1717 bp) consensus sequence using PhyML 3.0 (ATCG, Phylogeny.fr). The Gblocks programme was used for automatic alignment curation, while PhyML was used for tree building and TreeDyn programme was used to draw the phylogenetic tree [25–27]. All analyses are based on the maximum likelihood principal using an approximation of the standard Likelihood Ratio Test.

### Statistical analysis

Calculations regarding the prevalence of *Babesia* in red foxes from separate regions of Britain were performed only using data from animals where a location was known (253 / 316 samples). Comparisons of infection rates in relation to gender were made where data was available. All 316 samples were used when calculating the overall prevalence of *Babesia* DNA in red foxes in Britain.

Proportion positive (prevalence), with confidence intervals (95 % CI), was calculated for the overall study set as well as at regional level. Prevalence at the regional level was compared with the overall UK prevalence to determine if there was a significant difference (Minitab 15 (v15.1.0.0)). In addition the overall prevalence in male animals was compared with that in females (Minitab 15 (v15.1.0.0)). In these analyses, Fisher’s Exact Test was used where the number of events was less than 5; in all other cases the hypothesis test was based on the normal approximation.

## Results

### Screening of fox lung exudate samples for the presence of *Babesia* spp. DNA

Of the 120 samples initially screened, 29 samples gave positive results. From these nine samples that gave positive results for both duplicates from the semi-nested PCR (universal *Babesia*-*Theileria* primers BT1-F-BTH-1R and BT1-F-BT1-R) were cleaned as described above and sent for sequencing in one direction, using either the BT1-F or BTH-1 F primers. Following this initial screening all sequences returned with 99–100 % identity to several isolates of *Babesia annae* (previously referred to as: *Babesia* sp. ‘Spanish dog’ accession numbers EU583387, AY534602 and AF188001) [28]. No other *Babesia* species were identified in the fox lung exudate samples.

### Verification of PCR specificity

A random selection of nine positive samples from across Britain that gave positive results for both duplicates, were sent for sequencing using the *Babesia annae* specific BTFox1F and BTFox1R primers, this created a 597 bp consensus sequence (submitted to GenBank accession number KT580786). When this resultant sequence was compared using NCBI BLAST to the published sequences, it was found to have 100 % identity to several isolates of *Babesia annae* (previously referred to as: *Babesia* sp. ‘Spanish dog’ (accession numbers EU583387, AY534602 and AF188001) [28]. A further 13 positive samples were sequenced only using the BTFox1F primer; all of these sequences were 100 % identical to *Babesia annae* (previously referred to as: *Babesia* sp. ‘Spanish Dog’-AY534602, EU583387 and AF188001).

Seven clones were created from two of the positive samples (F501 (4 clones) and F340 (3 clones)) using the primers BT1-F & BT-Outer-R and BT1-F & BT-Inner-R. These clones were used to create two consensus sequences, which were identical to each other. One of the 1717 bp consensus sequences was submitted to GenBank (accession number KT580785). When this consensus sequence was compared using BLAST it demonstrated a 100 % identity to the published sequences of *Babesia annae* (previously referred to as: *Babesia* sp. ‘Spanish dog’ (accession numbers AY534602, EU583387 and AF188001).

### Distribution of *Babesia annae* in red foxes in Great Britain

Of the 316 lung exudate samples tested by nested PCR (using the BTFox1F and BTFox1R primers), 46 (14.55 % with a 95 % Confidence Interval (CI) of 10.66–18.44 %) returned positive results for the presence of *Babesia annae* (previously referred to as: *Babesia* sp. ‘Spanish dog’) DNA. Positive samples were found in foxes widely distributed across Great Britain (see Table 3), with *B. annae* DNA being found in Scotland as well as across the whole of England. The central region of England had the highest prevalence with 18 / 49 positive samples (36.7 % prevalence - 95 % CI 23.23 %–50.23 %), which was significantly (*p* = 0.007) higher than the British average. Of the regions of Britain where positive samples were found, Scotland had the lowest prevalence with only 6 / 80 positive samples (7.5 % prevalence - 95 % CI 1.72 %–13.27 %). Scotland had a significantly lower (*p* = 0.012) prevalence than the British average. No positive samples were found in Wales, though only 12 samples were available for analysis for this region.

**Table 3:** Prevalence of *Babesia annae* DNA in red foxes (*Vulpes vulpes*) from across Great Britain

Region	N^o^ tested	N^o^ Positive	Prevalence (%)	95 % CI	Gender	N^o^ / N^o^ Positive	% Prevalence	95 % CI
Scotland	80	6	7.5^*^	1.7–13.3 %	Male	48 / 5	10.4	1.8–19.0 %
					Female	32 / 1	3.1	0.00–9.2 %
Wales	12	0	0	-	Male	5 / 0	0	-
					Female	7 / 0	0	-
Northern (England)	34	8	23.5	9.3–37.8 %	Male	20 / 6	30	9.9–50.1 %
					Female	14 / 2	14.2	0.0–32.6 %
Central (England)	49	18	36.7^**^	23.2–50.2 %	Male	22 / 10	45	24.6–66.3 %
					Female	27 / 8	29.6	12.4–46.9 %
Southern (England)	78	11	14.1	6.4–21.8 %	Male	45 / 8	17.8	6.6–28.9 %
					Female	9 / 3	9.1	0.0–18.9 %
				Total	Male	140 / 29	20.7	14.0–27.4 %
					Female	113 / 14	12.4	6.3–18.5 %

*N* Number, *CI* Confidence interval

^*^ Significantly lower prevalence than nation average (*p* = 0.045)

^**^ Significantly higher prevalence than national average (*p* = 0.003)

The data shows that a higher number male foxes tested positive for parasite DNA than females in all the regions where positive samples were found (Table 3), although no statistically significant differences (*p* = 0.093) were observed between numbers of positive males and females when comparing genders across Britain.

### Phylogenetic relationship of *Babesia annae* and related species

**Fig. 1:**
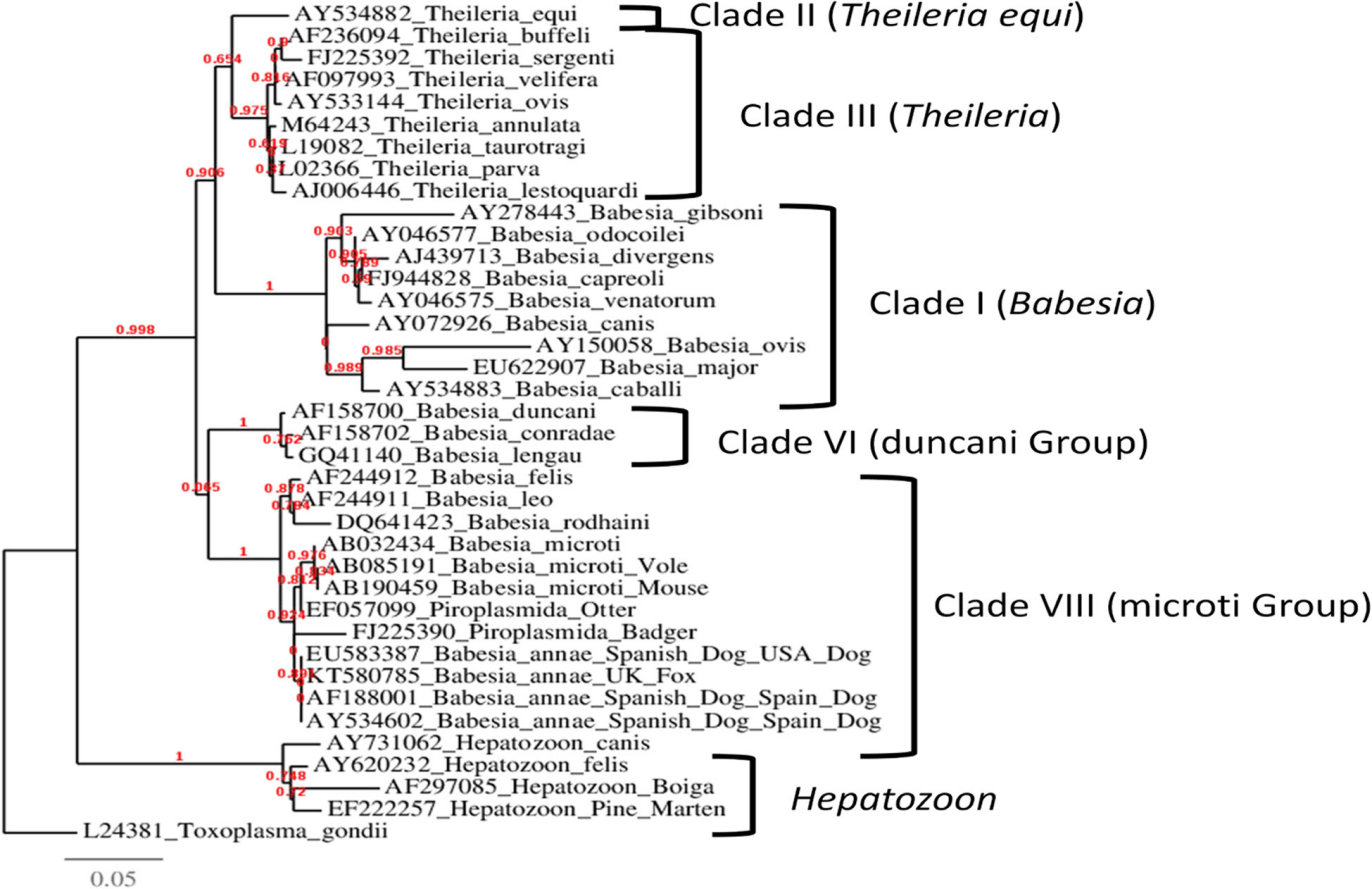
Phylogenetic analysis on 18S rRNA gene. Phylogenetic tree showing maximum likelihood approximation of the standard likelihood ratio test scores. A maximum likelihood approximation of the standard likelihood ratio test score of 0 indicating that no base pair substitutions were observed between AY534602, EU583387, AF188001 and KT580785. The phylogenetic analysis places KT580785 in clade VIII (microti group according to the nomenclature suggested by Lack and colleagues [26]).

Following maximum likelihood analysis it can be seen that *Babesia* parasites appear to be in three separate clades (Fig. 1) using the nomenclature suggested by Lack and colleagues [29], with KT580785 (*B. annae*) situated in clade VIII (microti group) with AY534602, EU583387 and AF188001, which are other published sequences of *Babesia annae* (previously referred to as: *Babesia* sp. ‘Spanish dog’) (see Fig. 1) found in Spain and the USA. The next most closely associated group of sequences have all been classified as *B. microti* (AB032434, AB085191 and AB190459); these were isolated from a human, bank vole and forest mouse respectively. Two *Piroplasmida* species found in Eurasian badges (*Meles meles*) (EF057099) and Eurasian otter (*Lutra lutra*) (FJ225390) were also closely related to *B. annae*. The remaining *Babesia* species are further separated into 2 clades: The classical *Babesia* group (clade I) and the duncani group (clade IV). The *Theileria* species, with the exception of *T. equi* are situated in clade III, while the *Hepatozoon* parasites are situated in a separate clade.

## Discussion

The results described in the current study clearly demonstrate that *B. annae* DNA is present widely in the red fox (*Vulpes vulpes*) population in Great Britain, with positive samples being found across all regions of England and throughout Scotland. Though we did not find any positives in the samples collected from Wales, the sample size for this region was small, with only 12 samples available for examination.

The overall prevalence of *B. annae* DNA from foxes in Britain was 14.6 % (see Table 1), the highest prevalence of *B. annae* was found in the central region of England where an overall prevalence of 36.7 % was observed, which was significantly (*p* = 0.007) higher than the national average. These figures are higher than the levels seen in foxes in numerous other European countries. In Croatia, 5.2 % (10 / 191) of foxes studied were PCR positive for *Babesia annae* (Synonym *Theileria annae*) DNA [13], while in Poland only 1 /123 (0.7 %) of fox spleens tested were PCR positive for *Babesia annae* (Synonym *Babesia microti*-like) DNA [14]. In two separate studies in Spain, 20 % (1 / 5) of foxes tested in the Burgos Region were positive for *Babesia annae* (Synonym *Theileria annae*) DNA [30], while in another study 50 % (5 / 10) of foxes from central Spain (Guadalajara) were positive for *Babesia annae* (Synonym *T. annae*, which had a 100 % identity to AF188001-*Babesia* sp. ‘Spanish Dog’) [23]. However these results may not reflect the actual prevalence across Spain, as sample numbers for both of these studies were very small with only 5 and 10 animals being tested, respectively. While a higher prevalence of *Babesia annae* (Synonym *Babesia microti*-like) has been found in Portugal where 63 / 91 (69.2 %) red foxes were PCR positive for the parasite [12].

An interesting observation from our study was that male foxes had a higher prevalence of *B. annae* DNA than females in all regions where positive samples were found (Table 3), though these differences were not statistically significant (*p* = 0.093). This may be a consequence that male foxes leave or are driven out of their home range territories in search of new territories, food and potential mates [31], increasing the chance of male foxes being exposed to infected ticks. Over recent years *Ixodes* tick numbers and their distribution appear to have been steadily increasing in Britain [32]. Exposure of foxes to *Babesia* parasites is likely to be through contact with infected ticks that occurs whilst scavenging and from small prey animals such as rodents and other small mammals, which are known to be reservoirs for *Babesia* parasites and also hosts to both common types of Ixodid ticks found in Britain (in particular *I. ricinus*). In a recent study in Britain, Brown and colleagues [33], demonstrated *B. microti* infections in 30.3 % of common shrews (*Sorex araneus*) and 30.4 % of field voles (*Microtus agrestis*) tested. Both shrews and voles were also found to be infested with *I. ricinus* ticks, strongly suggesting a role for these small mammals in the epidemiology of tick borne infections. We are unsure whether rodents can be infected with *B. annae*, as the parasite has never been described in any species other than canids. The primers used by Brown and colleagues [33] were *B. microti* specific and would not have detected *B. annae* even if it had been present. However *Babesia annae* (synonym *Theileria annae*) DNA has been demonstrated in both *I. ricinus* and *I. hexagonus* ticks in Spain [11, 19] with one tick being removed from a wood mouse (*Apodemus sylvaticus*). Alhough the tick tested positive for *Babesia annae* (synonym *Theileria annae*) DNA there is no evidence that the parasite was transmitted from the host mouse, or whether the tick was already infected prior to it attaching to the mouse [19]. There is also currently no evidence to prove if either of these species of ticks are competent vectors for the transmission of *B. annae* [34].

During this study we only detected parasite DNA from frozen exudates, we did not detect viable parasites or manage to examine blood smears for the presence of intra-erythrocytic life cycle stages. Nor was any clinical data available for the animals involved in this study, so we are unsure whether *B. annae* caused clinical symptoms in infected foxes or if it caused an asymptomatic infection. However, recent studies in Spain, Sweden and USA have demonstrated that *B. annae* is the causative agent of severe clinical disease and pathological abnormalities in dogs [1, 9, 34]. Clinical *Babesia* infections in dogs are often attributed to *B. canis* as there are few diagnostic tools for veterinarians to distinguish between the blood-borne piroplasms in routine veterinary practices [34], other than the direct examination of red blood cells under light microscopy. In laboratories the immunofluorescent antibody test (IFAT) is used for serological diagnosis, while PCR generally corresponds to a more sensitive and specific diagnostic tool.

Our phylogenetic analysis shows that the 18S rRNA gene of *B. annae* described in this study is identical to that described in Europe with a maximum likelihood approximation of the standard likelihood ratio test score of 0 indicating that no base pair substitutions were observed between AY534602, EU583387, AF188001 and KT580785. The sequences generated also showed a closer parsimony to *B. rodhaini, B. felis* and *B. leo* than *B. divergens, B. gibsoni* and *B. duncani*. The sequence analysis carried out in this study agrees with that carried out by Lack and colleagues [29], who placed *B. microti* and the *microti*-like parasites in a distinct phylogenetic clade (referred to as the microti Group) which included *Babesia* ‘Spanish dog’ and *B. microti* found in mice (AB190459) and bank voles (AB085191) amongst others [29]. The phylogenetic data presented in this study shows that the *Babesia* are separated into three distinct clades, while the *Theileria* and *Hepatozoon* species are also all situated in separate clades. However the piroplasmida parasites found in otters and badgers (EF057099 and FJ225390) are positioned in between the *Babesia* clades, suggesting these members of the phylum require further reclassification.

More work needs to be carried out to help determine the dynamics of transmission of *B. annae* to foxes; an examination of small prey animals (rodents etc.) and the ticks that infest them may help demonstrate their role in maintaining *B. annae* infections in the environment. Further studies are also required to examine cases of canine babesiosis in Britain to speciate the causative agent and determine if *B. annae* is present within the British dog population and if it is causing clinical disease in canids.

## Conclusions

This is the first study to demonstrate the presence of *B. annae* DNA in Britain. Sequence analysis has shown the *B. annae* 18S rRNA gene sequence detected in foxes in Britain to be identical to that detected in foxes in Europe and North America. Phylogenetic analysis shows that *B. annae* is closely related to *Babesia microti*, but clearly is a distinct species.
