# Erratum to: Detection of *Babesia annae* DNA in lung exudate samples from Red foxes (*Vulpes vulpes*) in Great Britain

**DOI:** 10.1186/s13071-016-1570-x

**Published:** 2016-05-11

**Authors:** Paul M. Bartley, Clare Hamilton, Cari Wilson, Elisabeth A. Innes, Frank Katzer

**Affiliations:** Moredun Research Institute, Pentlands Science Park, Bush Loan, Midlothian, EH26 0PZ Scotland UK

Unfortunately, we noticed that the reference list of our published paper [1] contains an error in the title of reference 8. The corrected reference is:

8. Baneth G, Florin-Christensen M, Cardoso L, Schnittger L. Reclassification of *Theileria annae* as *Babesia vulpes* sp. nov. Parasit Vectors. 2015;8:207.

We would like to apologize for this error and for any inconvenience this may have caused.
